# A cross sectional evaluation of a total smoking ban at a large Australian university

**DOI:** 10.1186/s13104-016-2090-7

**Published:** 2016-05-26

**Authors:** Sharyn Burns, Ellen Hart, Jonine Jancey, Jonathan Hallett, Gemma Crawford, Linda Portsmouth

**Affiliations:** Collaboration for Evidence, Research and Impact in Public Health, School of Public Health, Curtin University, GPO Box U1987, Perth, WA 6845 Australia

## Abstract

**Background:**

Total smoking bans have been found to contribute positively to the health of non-smokers by reducing exposure to second-hand smoke, and to enhance the likelihood of cessation among smokers.

**Methods:**

Two cross-sectional electronic surveys of staff and students at a large Australian university were conducted prior (n = 969) and 1 year post (n = 670) the implementation of a smoke free campus policy. Demographics, tobacco use, intention to quit, attitudes towards smoking and smoking restrictions and awareness of and attitudes towards the campus smoking policy were measured.

**Results:**

Exposure to second-hand smoke (SHS) reduced significantly (p < 0.001) one year after policy implementation. Smoking prevalence was similar at both time periods (T1 9.3 %; T2 8.4 %) and over half of smokers indicated they were planning to quit smoking in the future (T1 65.5 vs T2 62.3 %). There was a significant increase in positive responses to the statement *the campus should be totally smoke free including all outdoor areas* at T2 compared to T1 (T1 60.8 vs T2 71.4 %; p < 0.001), however respondents felt there should be places on campus for smokers to smoke (T1 53.6 vs T2 47 %; p < 0.05).

**Conclusions:**

This study found a significant positive difference in exposure SHS after implementation of the total ban. Although prevalence of smoking in this study was low, the proportion of respondents who were contemplating smoking cessation suggests support for smokers would be beneficial. Continued awareness raising, education and enforcement is likely to enhance the long term outcomes of the total ban.

## Background

Australia has employed a coordinated, comprehensive approach to tobacco control since the 1980s [1, 2] and as a result has experienced a significant decline in the social acceptability of tobacco use and smoking prevalence [3]. Despite substantial progress in reducing smoking prevalence and exposure to second-hand smoke (SHS), tobacco smoking remains the largest contributor to and one of the most preventable causes of ill-health in Australia [4].

One strategy employed as part of a comprehensive approach has been the introduction of smoke-free policies (prohibiting smoking in specific areas), which have been found to contribute to reductions in smoking rates and in turn SHS exposure and improved health outcomes [5, 6]. Australia has a long history of smoke-free policy with restrictions commencing in the 1970s [7] and by 2002 most enclosed public spaces were smoke-free in almost all Australian states and territories [7]. By 2010, smoking was banned in all non-hospitality workplaces, restaurants and bars in all jurisdictions in Australia [5]. Some high income countries, including Australia have extended smoke-free policies to outdoor spaces [5] consistent with Western Australian legislation smoking at this university was prohibited in all enclosed areas and cafes [8], however in 2012 the ban was extended to include all outdoor areas and university vehicles. Although US studies have demonstrated favourable changes in smoking behaviour [9] and exposure to SHS [10] as a result of a total smoke free policy implementation, there is a paucity of data describing the evaluation of total smoking bans in universities in Australia.

Most Australians are aware of the harms of smoking and SHS; reflected by negative normative attitudes towards smoking [3]. These changes in attitudes over the last few decades have coincided with the implementation of smoke-free policies and declining prevalence of smoking rates [3, 7]. Smoke-free policies have been associated with reduced respiratory symptoms, improved lung function, and improved cardiovascular health for people who would have previously been exposed to SHS [11]. A study of the impact of tobacco bans in 15 Ontario municipalities found exposure to SHS decreased by 4.7 % and 2.3 % in public places and workplaces respectively over a 2 year period. The same study suggested these bans also reduced exposure to SHS in private settings including homes and cars [12].

While smoke-free policies assist in improving the health of non-smokers they also appear to impact on the health of smokers by reducing the amount of cigarettes smoked per day and to support motivation for cessation [5]. A US study found smoke-free laws (which included bans in at least one location including workplaces, restaurants and bars) were associated with decreased current and established smoking, but not with past year initiation among young adults [13]. Others have found total smoking bans to be more likely to promote quit attempts compared to partial smoking bans [14]. A study in California found smokers living in a city with a smoking ban in outdoor spaces were more likely to reduce smoking levels and to attempt to quit compared to those living in other cities [15]. A qualitative study of smokers at this university found some smokers felt the implementation of the total ban would provide good motivation for them personally to quit [16].

Policy support is a key factor for successful implementation; Australians, including smokers, are generally supportive of smoke-free policy [5, 17, 18]. Arguments used by opponents of smoke-free areas (e.g. the difficulty of enforcement and the risk that policies may be economically detrimental to specific industries, particularly the hospitality industry) have been unfounded [5]. Although only short term compliance has been measured, policy compliance is generally high in most countries, and studies assessing the impact of smoke-free policy implementation have found no changes to hospitality revenue [5]. Despite strong support for such policy in enclosed areas, extensions to include outdoor spaces may be met with greater resistance [5]. Support for smoking bans at a range of community venues including zoos, parks and community events has been found to be strongest for smaller outdoor venues. Women with children were more supportive of total bans than other respondents [19]. While overall support for total smoking bans in universities has been positive, both smokers and non-smokers have recognised the rights of smokers and suggested campuses provide designated places for smokers to smoke [10, 20].

A total smoking ban (including all buildings, grounds, outdoor areas, student housing and university vehicles) was implemented in a large university campus in Western Australia. Prior to this ban, smoking was prohibited in all buildings and undercover areas of the university. The university presents particular challenges for the implementation of a total ban due to the physical size of the ground (119 hectares, which includes student housing in addition to extensive outdoor areas including gardens, sports fields and natural bushland). In 2012 more than 35,000 students were located at the Bentley campus. Of the total student population approximately 80 % were undergraduate students and 55 % were female. Across all campuses the university employed 1378 academic and 1709 general staff (not including causal staff) of whom 54 % were female. This paper reports on exposure to SHS, attitudes towards smoking, and awareness of and attitudes towards the policy according to smoking status at baseline and one year following implementation.

## Methods

Two cross-sectional electronic surveys of staff and students were conducted, prior to the smoke free campus policy implementation (T1) and 1 year post policy implementation (T2). For each survey a random sample of students (n = 4500) and staff (n = 500) were invited to participate via email. Participants at T1 and T2 were matched to ensure samples were independent. Three participants who completed the survey on both occasions were excluded from the T2 analysis.

The use of a cross-sectional sample is often used in the exploration of attitudes towards and health behaviour compliance and policy implementation [21–24]. Surveys were sent from the University Surveys Office to participants meeting the inclusion criteria: aged over 18 years; staff working at the main campus or students enrolled internally and attending the main campus. Students were sent two and staff one follow-up reminder email. Reminder emails were kept to a minimum to reduce burden on staff and students and were limited due to project resources.

### Instrumentation

The survey instrument collected data on demographics, tobacco use, attitudes towards smoking and smoking restrictions, awareness of and attitudes towards campus smoking policy and intentions to quit. All questions were developed based on previously validated instruments [17]. To ensure the survey was appropriate for the target audience it was tested for reliability using a test–retest (n = 32), and for content validity using an expert panel of health promotion, research, and tobacco control professionals (n = 8). Items with low internal consistency were removed or modified [17].

### Demographics

Demographics included gender and primary role at the university. Primary role asked respondents to identify their main role at the university from four options: undergraduate student, postgraduate student, academic staff or general/professional (administrative/technical) staff [17].

### Tobacco use and second-hand smoke exposure

Similar to other university-based studies [25, 26] four categories of smoking status were defined: non-smoker (never smoked cigarettes or never smoked regularly); ex-smoker (previously smoked regularly-at least one cigarette a day); regular smoker (at least one cigarette a day); or occasional smoker (less than one cigarette a day on average). For the purpose of comparison with key independent variables the regular and occasional smoker variables were collapsed to form one variable (smoker). SHS exposure was measured by self-reported exposure to cigarette smoke on campus in the previous four weeks [17, 25, 27].

### Attitudes towards smoking and smoking restrictions

Questions on attitudes towards smoking were adapted from previously validated questions [27]. Attitudes towards a smoke-free campus were measured using questions adapted from a study of staff at Australian TAFEs (technical and further education—tertiary institutions offering a variety of vocational education and training) [28]. Response options included agree, neutral, and disagree [17].

### Awareness and attitudes towards campus smoking policy

Respondents were asked if they were aware of a campus smoking policy that restricted smoking on campus (response options: yes, no, don’t know/not sure) and subsequently how they would describe the current campus smoking policy. Attitudes towards the impact of a smoke-free campus were measured by four items: staff quality of life; student quality of life; student learning; and student enrolment with three responses: negative, neither negative nor positive, and positive.

### Data analysis

Data was analysed using SPSS for Windows version 20.0. The dependent variable was tobacco use; independent variables included exposure to SHS, attitudes towards smoking, attitudes towards smoking bans, and awareness of campus smoking policy. Demographics of the sample were explored through descriptive statistics. Chi square analysis was used to explore the impact of a smoke-free campus for smokers, ex-smokers, and non-smokers. Due to the cross sectional nature of the study z-tests were calculated to explore any differences between the baseline and post samples [9]. Differences were considered significant at p < 0.001 and moderately significant at p < 0.05.

## Results

Of the staff and students who were invited to participate, 969 respondents (62.6 % female) provided complete data at T1 and 670 (64.6 % female) at T2. This represented a response rate of 19.4 % at T1 and 13.4 % at T2.

There was no significant difference between gender, length of smoking or plans to quit smoking at T1 and T2. Use of tobacco was similar although there was a moderately significant difference in respondents who had previously smoked with T2 respondents being more likely to categorise themselves as an ex-smoker (T1 11 vs T2 14.7 %; p < 0.05) (see Table 1). Smoking prevalence was similar at both time periods with 9.3 and 8.4 % of respondents reporting to smoke at T1 and T2 respectively. Over half of smokers indicated they were planning to quit smoking in the future (T1 65.5 vs T2 62.3 %). There was a significant difference in primary role at the University with more undergraduate students responding at baseline (61.8 %) compared to T2 (54.3 %) and a greater proportion of general/professional staff responding at T2 compared to T1 (T1 13.6 vs T2 17.6 %). Demographics of respondents are described in Table 1.

**Table 1:** Demographics and smoking status at T1 and T2

	T1	T2	Significance (p) T1/T2
Gender			
Male	362 (37.4)	237 (35.4)	0.412
Female	607 (62.6)	433 (64.6)	0.412
Total	969	670	
Primary role			
Undergraduate student	599 (61.8)	364 (54.3)	0.002**
Postgraduate student	141 (14.6)	108 (16.1)	0.384
General/Professional staff member	132 (13.6)	118 (17.6)	0.027*
Academic staff member	97 (10)	80 (11.9)	0.215
Total	969	670	
Use of tobacco			
Never smoked cigarettes at all, or never smoked them regularly (non-smoker)	771 (79.6)	508 (76.2)	0.101
Do not smoke now but used to smoke them regularly (once or more per day) (ex-smoker)	107 (11)	98 (14.7)	0.029*
Occasionally smoke (on average, less than one per day) (smoker)	39 (4)	34 (5.1)	0.303
Currently smoke cigarettes (more than one per day) (smoker)	51 (5.3)	22 (3.3)	0.059
Prefer not to answer	1 (0.1)	5 (0.7)	0.033*
Total	969	667	
Are you planning on quitting smoking?			
Yes	59 (65.5)	38 (62.3)	0.682
No	31 (34.5)	23 (37.7)	0.001*
Total	90	61	

* p < 0.05, ** p < 0.01

### Second-hand smoke exposure

Although respondents reported some exposure to SHS at both T1 (79.4 %) and T2 (58.1 %), levels of exposure fell significantly between the two time periods (p < 0.001). Approximately one-third of respondents (33.2 %) reported no exposure to SHS while on campus during the past four week period at T2 compared to 15.2 % at T1 (p < 0.001). At T1 23.8 % of respondents reported to have been exposed to SHS once or more daily and 34.8 % at least once a week compared to 8.6 and 21.4 % respectively at T2 (p < 0.001) (Table 2). At both time periods most respondents agreed that *second*-*hand smoke causes harm* (T1 84.1 vs T2 85.1 %) however smokers were less likely to agree with this statement compared to the total population (T1 46.7 vs T2 42.6 %).

**Table 2:** Agreement with tobacco smoking attitude statements reported by University staff and students at T1 and T2

Total sample Baseline (n = 968) Post (n = 636)	Non-smokers N (%)	Ex-smokers N (%)	Smokers N (%)	Total # of participants who agree N (%)
Total				
Baseline (T1)	771	107	90	
Post (T2)	489	93	54	
If someone smokes cigarettes around me they are causing me harm because of second-hand smoke				
Baseline	686 (89)	86 (80.4)	42 (46.7)	814 (84.1)
Post	441 (90.2)	77 (82.8)	23 (42.6)	541 (85.1)
Significance (T1/T2)	0.497	0.66	0.631	0.596
I prefer to socialise in a smoke-free environment				
Baseline	695 (90.1)	86 (80.4)	30 (33.3)	811 (83.8)
Post	445 (91)	75 (80.6)	21 (38.9)	541 (85.1)
Significance (T1/T2)	0.610	0.960	0.502	0.490
I seek out smoke-free environments				
Baseline	572 (74.2)	67 (62.6)	13 (14.4)	652 (67.4)
Post	388 (79.3)	60 (64.5)	11 (20.4)	459 (72.2)
Significance (T1/T2)	0.037*	0.779	0.358	0.041*
It disappoints me when a friend who normally doesn’t smoke, smokes cigarettes while drinking				
Baseline	547 (70.9)	46 (43)	13 (14.4)	606 (62.6)
Post	364 (74.4)	40 (43)	9 (16.7)	413 (64.9)
Significance (T1/T2)	0.177	1	0.719	0.342
I would rather date a non-smoker				
Baseline	701 (90.9)	84 (78.5)	36 (40)	821 (84.8)
Post	458 (93.7)	74 (79.6)	18 (33.3)	550 (86.5)
Significance (T1/T2)	0.080	0.857	0.424	0.352
I ask others not to smoke around me				
Baseline	354 (44.7)	28 (26.2)	2 (2.2)	375 (38.7)
Post	231 (47.2)	39 (41.9)	6 (11.1)	276 (43.4)
Significance (T1/T2)	0.646	0.018*	0.024*	0.062

* p < 0.05, ** p < 0.01

### Attitudes towards smoking

When all responses were considered, attitudes towards smoking were generally negative with little change in attitudes between T1 and T2 (see Table 2). Most respondents agreed that they would *prefer to socialise in a smoke*-*free environment* (T1 83.8 vs T2 85.1 %) and they would *prefer to date a non*-*smoker* (T1 84.8 vs T2 86.5 %). A greater proportion of respondents suggested they *would seek out smoke*-*free environments* at T2 compared to T1 (T1 67.4 vs T2 72.2 %; p < 0.05). For both time periods smokers reported more positive attitudes towards smoking compared to non-smokers and ex-smokers. Ex-smokers were significantly more likely to agree that *second*-*hand smoke causes harm* (T1: 80.4 vs T2: 82.8 %; p < 0.05) and to agree that they would *ask others around them not to smoke* at T2 compared to T1 (T1 26.2 vs T2 41.9 %; p < 0.002).

### Attitudes towards smoke-free campus

There was strong agreement that the campus should be smoke-free in all buildings at both time periods (T1 91.3 vs T2 92.8 %). Although there was less agreement that *the campus should be totally smoke*-*free including all outdoor areas* there was a significant increase in positive responses to this statement at T2 compared to T1 (T1 60.8 vs T2 71.4 %; p < 0.001). This was true for non-smokers (p = 0.017) and ex-smokers (p = 0.014) however there was no statistical significance in responses for smokers between the two time periods (p = 0.562) (see Table 3). Respondents were significantly more likely to agree that *restrictions on where you can smoke make it hard for smokers on campus* at T2 compared to T1 (T1 38.5 vs T2 56.6 %; p < 0.01). Agreement with the statement at T2 was more likely for both non-smokers (p < 0.001) and smokers (p < 0.05). Non-smokers were less likely to agree that *there should be some places on campus where people can go to smoke* at T2 compared to T1 (T1 48.9 vs T2 42.3 %; p < 0.05), however there were no significant differences in attitudes towards this statement for ex-smokers or smokers between the two time periods.

**Table 3:** Agreement with tobacco control attitude statements reported by University staff and students at T1 and T2

Total sample Baseline (n = 968) Post (n = 636)	Non-smokers N (%)	Ex-smokers N (%)	Smokers N (%)	Total # of participants who agree N (%)
Total				
Baseline (T1)	771	107	90	
Post (T2)	489	93	54	
Our campus should be smoke-free including all out door areas				
Baseline	517 (67.1)	54 (50.5)	18 (20)	589 (60.8)
Post	378 (77.3)	63 (67.7)	13 (24.1)	454 (71.4)
Significance (T1/T2)	0.000**	0.014*	0.562	0.000**
The restrictions on where you can smoke makes it hard for smokers on campus				
Baseline	275 (35.7)	54 (50.5)	44 (48.9)	373 (38.5)
Post	262 (53.6)	58 (62.4)	40 (74.1)	360 (56.6)
Significance	0.000**	0.091	0.003**	0.000**
There should be some places on campus where people can go to smoke				
Baseline	377 (48.9)	72 (67.3)	70 (77.8)	519 (53.6)
Post	207 (42.3)	54 (58.1)	38 (70.4)	299 (47)
Significance (T1/T2)	0.023*	0.177	0.322	0.009**
There should be more help or support at university for people who want to quit smoking				
Baseline	470 (61)	59 (55.1)	43 (47.8)	572 (59.1)
Post	295 (60.3)	40 (43)	18 (33.3)	353 (55.5)
Significance (T1/T2)	0.826	0.087	0.891	0.156
Because of their professional role, university staff have a responsibility to be non-smokers				
Baseline	280 (36.3)	20 (18.7)	8 (8.9)	308 (31.8)
Post	191 (39.1)	22 (23.7)	8 (14.8)	221 (34.7)
Significance (T1/T2)	0.327	0.39	0.271	0.223
Our campus should be smoke free in all buildings				
Baseline	724 (93.9)	97 (90.7)	63 (70)	884 (91.3)
Post	467 (95.5)	88 (94.6)	35 (64.8)	590 (92.8)
Significance (T1/T2)	0.226	0.289	0.516	0.298
Our campus should be completely smoke-free				
Baseline	553 (71.7)	63 (58.9)	20 (22.2)	636 (65.7)
Post	345 (70.6)	57 (61.3)	10 (18.5)	412 (64.8)
Significance (T1/T2)	0.653	0.726	0.596	0.704

* p < 0.05, ** p < 0.01

### Awareness of campus smoking policy

Awareness of campus smoke free policy increased significantly between T1 and T2 with 56 % of respondents reporting they were aware that there was a smoke free policy at T1 compared to 79.8 % at T2 (p < 0.001). However when this response was analysed by smoking status; awareness of the policy increased for non-smokers, ex-smokers and smokers, although changes were only significant for non-smokers and ex-smokers (p < 0.001). Awareness of specific aspects of the policy varied. At T1 58.7 % of respondents correctly identified that staff, students and visitors were allowed to smoke in designated areas of the campus but not inside the buildings. At T2, 65.9 % of respondents correctly identified that smoking was now banned throughout the campus (Table 4).

**Table 4:** Staff and students awareness of campus tobacco smoking policy at T1 and T2

Total Sample Baseline (n = 968) Post (n = 636)	Non-smokers N (%)	Ex-smokers N (%)	Smokers N (%)	Total # of participants who agree with the statement N (%)
Total				
T1	771	107	90	
T2	489	93	54	
Awareness of policy				
Yes				
Baseline	405 (52.5)	72 (67.3)	65 (72.2)	542 (56)
Post	396 (78.7)	83 (86.5)	43 (78.2)	522 (79.8)
Significance (T1/T2)	0.000**	0.000**	0.322	0.000**
No				
Baseline	189 (24.5)	12 (11.2)	19 (21.1)	220 (22.7)
Post	47 (9.3)	12 (12.5)	4 (7.3)	63 (9.6)
Significance (T1/T2)	0.000**	0.711	0.03*	0.000**
Don’t know/not sure				
Baseline	177 (23)	23 (21.5)	6 (6.7)	206 (21.3)
Post	60 (11.9)	1 (1)	8 (14.5)	69 (10.6)
Significance (T1/T2)	0.000**	0.000**	0.11	0.000**
Describe the policy				
No policy on tobacco smoking in place				
Baseline	43 (5.6)	4 (3.7)	3 (3.3)	50 (5.2)
Post	22 (4.4)	3 (3.1)	6 (10.9)	31 (4.7)
Significance (T1/T2)	0.401	0.842	0.062	0.697
Staff, students, and visitors are allowed to smoke tobacco in designated areas of campus buildings				
Baseline	65 (8.4)	9 (8.4)	13 (14.4)	87 (9)
Post	25 (5)	4 (4.2)	2 (3.6)	31 (4.7)
Significance (T1/T2)	0.026*	0.238	0.041*	0.001**
Staff, students, and visitors are allowed to smoke tobacco in designated areas of the campus grounds but not inside the buildings				
Baseline	447 (58)	65 (60.7)	56 (62.2)	568 (58.7)
Post	62 (12.3)	10 (10.4)	4 (7.3)	76 (11.6)
Significance (T1/T2)	0.000**	0.000**	0.000**	0.000**
Staff, students, and visitors are banned from smoking tobacco throughout the campus; this includes all university buildings grounds and vehicles				
Baseline	61 (7.9)	11 (10.3)	5 (5.6)	77 (8)
Post	324 (64.4)	69 (71.9)	38 (69.1)	431 (65.9)
Significance (T1/T2)	0.000**	0.000**	0.000**	0.000**
Don’t know/not sure				
Baseline	155 (20.1)	18 (16.8)	13 (14.4)	186 (19.2)
Post	70 (13.9)	10 (10.4)	5 (9.1)	85 (13)
Significance (T1/T2)	0.009**	0.219	0.363	0.001**

* p < 0.05, ** p < 0.01

## Discussion

### Second-hand smoke exposure

This study found significant reductions in all levels of exposure to SHS over the two time periods. Despite these promising results only one-third of respondents (33.2 %) reported to have never been exposed to cigarette smoke at T2 which indicates SHS exposure remains present and unacceptably high. Smoke-free policies may drive smokers to the periphery of campus and thereby still expose students and staff to SHS during entry and exit to campus [10]. This is especially pertinent to this university as the grounds are large (116 hectares) with poorly lit perimeters making policy enforcement challenging [17].

The finding that a proportion of people continue to smoke on campus despite the ban is supported by an observational study at this university which was implemented to measure compliance. Of the 50 smokers observed, 37 agreed to participate in an intercept survey. Reasons for noncompliance with the policy included defiance, necessity to smoke, inconvenience of travelling off campus, unintentional noncompliance, and ease of avoidance of detection, which may stem from inadequate enforcement [29]. Enforcement is a key component to supporting smoke-free policy and it is recommended that it is implemented with initial warnings and education, progressing to penalties if individuals continue not to comply with the policy [30, 31].

### Prevalence of tobacco smoking

Prevalence of tobacco smoking in this study was lower than for the general adult population in Australia (daily smoking 12.8 % in 2013) [32] however these findings are consistent with other studies conducted at this university [25]. The implementation of the smoke-free policy had little impact on the prevalence of smoking over the two time periods however this may be due to the low baseline prevalence and the short time period that the policy had been in place. Although studies have demonstrated success in reducing prevalence through the implementation of policy [9] it is also acknowledged that policy is best combined with other strategies [1, 33]. Over 60 % of smokers in this study indicated they were planning to quit smoking in the future. Consistent with the Transtheoretical Model which helps to explain different stages of change in the behaviour change process, smokers in the contemplation and preparation stages are ideal participants for quit smoking interventions [34].

### Attitudes to smoking

Attitudes towards tobacco smoking remained similar over the two time periods. Consistent with Australian data the majority of non-smokers reported negative attitudes towards smoking [3]. The lack of change may reflect the social norms associated with smoking in Australia [35] and/or the predominately policy based nature of the intervention as the policy implementation included minimal awareness raising strategies and quit sessions for staff.

Support for policy implementation has been identified as critical to success [36]. Similar to this study, US college studies have reported students to be generally supportive of smoke-free policy, especially smoke-free buildings [20, 37] and the reduction of SHS, however they were less likely to agree that the campus should be totally smoke-free [20]. Although the current study found around half of all respondents felt there should be places people can go to smoke on campus at both time periods this was lower (T1 53.6 vs T2 47 %; p < 0.05) at T2. As in this study, a US study found while smokers were more likely to agree that there should be some places on campus to smoke, non-smokers also felt smokers had the right to smoke on campus [20]. While 92.8 % of respondents in the current study felt the campus should be smoke-free in all buildings at T2 only 64.8 % felt the campus should be completely smoke-free. There was no significant difference in these responses over the two time periods. Similarly, another US study reported that over 70 % of college students and staff agreed that their campus should be totally smoke-free [10]. Consistent with other studies [10] the current study found smokers to be least supportive of smoke-free policy. Despite changing norms towards smoking, and support generally being shown for smoke-free policies by smokers, some studies still find this group to be unsupportive of smoke-free policies which may translate into non-compliance [17, 28].

While changes in policy awareness were positive between the two time periods, over one-third of respondents (34.1 %) were not aware of the specific restrictions associated with the total ban on smoking on campus at T2 supporting the need to continue to promote and enforce the policy. Successful implementation of smoke-free policy should include a comprehensive range of strategies (for example, awareness raising, education, quit sessions) and the policy needs to be consistently enforced [5, 17]. Other studies assessing the implementation of smoke-free policy in large learning institutions have found that enforcement can be difficult even when there is a perception of high compliance among the population [28, 38]. Staff and students from a New Zealand university suggest compliance should be enforced, potentially by campus security [31]. Similarly, key stakeholders and staff and student smokers from this university suggested enforcement to be an important consideration for the effectiveness of the policy, however given the extensive grounds of this university this would be challenging unless sufficient resources were provided [16, 17]. Currently the policy is enforced by Campus Security staff who issue a warning or fine to repeat offenders.

A successful smoke-free policy can educate the community on the health benefits of not smoking, and provide assistance for smoking cessation [17, 22, 39] however policy implementation needs to also consider the stigmatisation of smokers and in the case of this campus, smokers safety if they are to leave grounds to smoke [16, 29]. A qualitative study conducted at this university found smokers to feel stigmatised and marginalised [16]. While stigmatising smoking has been a strategy of smoking control programs [10] there has been less focus on the unintended consequences which may include guilt, poor self-esteem and continued maintenance of smoking [40]. It is recommended public health interventions consider the negative psychological consequences of stigmatization and include strategies that focus on positive reinforcement and cessation support [40].

Universities provide a challenging yet important setting for health promotion. For young university students time at university represents an important transitional period where health behaviours such as tobacco use may become established [25]. While universities provide a range of social experiences which have the potential to influence smoking initiation and maintenance [25, 41] they also provide an ideal setting to positively impact health behaviours, including tobacco use [25].

## Limitations

The cross sectional nature of this study precludes any causal effects. The low proportion of smokers who participated in this survey, while similar to other Australian university and TAFE [25, 28] and New Zealand university [31] studies may be due to selective non-reporting or under-reporting [26]. The low response rate, while similar to other studies [28, 42] is a limitation, however this study had limited resources for incentives and follow up. Although there are slightly more female students (55 %) and staff (54 %) at the university the overrepresentation of females (64.6 %) is also a limitation of this study. A higher proportion of smokers may have resulted in more positive attitudes towards smoking and more negative views of related policy.

## Conclusions

Despite minimal supporting strategies, the implementation of the total smoking ban on campus resulted in significant reductions in exposure to second-hand smoke on a daily and weekly basis. While smoking prevalence did not change during the one year time period this was low at T1 (9.3 %). The study found strong support for the policy, however as expected smokers were least likely to support the total ban. The findings demonstrate the benefits of total smoking bans in large institutions but also highlight the need for a range of coordinated strategies which include awareness raising, education and enforcement, with targeted intervention such as cessation support available for those who smoke.

## Abbreviations

SHS: second-hand smoke
T1: data collection: time period 1
T2: data collection: time period 1
